# Menopausal symptoms and quality of life in female survivors treated with hematopoietic stem cell transplantation

**DOI:** 10.3389/fpsyt.2023.1050959

**Published:** 2023-02-28

**Authors:** Huina Su, Huiling Li, Hua Zhang, Xin Yang, Chaohua Wang

**Affiliations:** ^1^Department of Obstetrics and Gynecology, Peking University People's Hospital, Beijing, China; ^2^Clinical Epidemiology Research Center, Peking University Third Hospital, Beijing, China

**Keywords:** hematopoietic stem cell transplantation, menopausal symptoms, quality of life, female survivors, SF-36

## Abstract

**Objectives:**

To assess the severity of menopausal symptoms and the correlation among different quality of life questionnaires and compare the quality of life of patients who underwent hematopoietic stem cell transplantation (HSCT) for hematological disorders with the norm group in order to facilitate personalized and directed therapeutic intervention for patients.

**Methods:**

We recruited women who had premature ovarian failure (POF) after HSCT for hematologic diseases in the gynecological endocrinology outpatient clinic of Peking University People's Hospital. Women with HSCT were included in the study if they had 6 months of spontaneous amenorrhea with serum follicle-stimulating hormone levels greater than 40 mIU/mL taken 4 weeks apart. The patients who had other causes of POF were excluded. During the survey, all women were required to fill out the questionnaires [Quality of Life Questionnaire (MENQOL), Generalized Anxiety Disorder-7 (GAD-7), Patient Health Questionnaire-9 (PHQ-9), and 36-item Short-Form (SF-36)] online. We analyzed the severity of menopausal symptoms, anxiety, and depression in Participants. In addition, differences on the SF-36 scale scores between the study group and norm groups were examined.

**Results:**

In total, 227 (93.41%) patients completed the survey and were analyzed. The severity of all symptoms is “none and mild” in MRS, MENQOL, GAD-7, and PHQ-9. On the MRS, the most common symptoms were irritability, physical and mental exhaustion, and sleep problems. The severest symptoms were sexual problems (53, 73.82%), followed by sleep problems (44, 19.38%) and mental and physical exhaustion (39, 17.18%). In the MENQOL, the most common symptoms were psychosocial and physical symptoms. The severest symptoms were sexual symptoms (35, 48.75%) followed by psychosocial symptoms (23, 10.13%). Moderate-severe scores were shown in 11.89% (27) and 18.72% (42) cases in the GAD-7 and PHQ-9, respectively. Based on SF-36, in comparison with the norm group, the HSCT participants had higher vitality scores and lower role physical, physical functioning, and role emotional scores aged 18–45. In addition, the HSCT participants had lower mental health scores aged 18–25, and lower general health scores aged 25–45. No strong correlation was observed between questionnaires in our study.

**Conclusion:**

Overall, menopausal symptoms are milder in female patients after HSCT. There is no single scale that comprehensively assesses the patient's quality of life after HSCT. We need to assess the severity of various symptoms in patients using different scales.

## 1. Introduction

Hematopoietic stem cell transplantation (HSCT) is a rapidly evolving technique for treating hematological malignancies such as acute myeloid leukemia (AML), acute lymphoblastic leukemia (ALL), chronic myeloid leukemia (CML), Hodgkin lymphoma (HL), non-Hodgkin lymphoma (NHL), and non-malignant hematological disorders, including aplastic anemia, thalassemia, and myelofibrosis. HSCT is also used for solid tumors that respond to chemotherapy, such as some germ cell tumors. By increasing the success rate of HSCT, the number of HSCT survivors in the United States alone is expected to exceed half a million by 2030, and 14% of them will be children less than 18 years of age (1). In 2017, the Chinese Blood and Marrow Transplantation Registry Group (CBMTRG) published a report on transplant activity between 2008 and 2016 in China. The report showed a continued increase in HSCT, especially in Haploidentical donors. In 2020, the annual number of HSCTs in China was more than 10,000 for the first time (2). In China, 42% of the HSCT patients were female, and 58% were male in 2019. The number of adolescent patients (≤18 years of age) was 3,752, and 96% of them underwent allogeneic HSCT (3). HSCT survivors often have higher risks of gynecologic complications, such as abnormal uterine bleeding, amenorrhea, premature ovarian insufficiency (POI), fertility preservation, vulvovaginal graft-versus-host disease (GVHD), and cervical cancer screening, HPV vaccination, and sexual complaints were more common among them. Sixty-five to eighty-six percent of transplant recipients have been observed with premature ovarian failure (POF) after HSCT (4). This incidence of POF rises up 100% among patients who receive myeloablative conditioning regimens before HSCT composed of high-dose chemotherapy (5, 6). With the number of long-term survivors of HSCT, reports of late complications in the physical, psychological, and social dimensions have increased. Late side effects of HSCT are various. Most complications are not life-threatening but still profoundly affect the quality of life in long-term survivors (7). Although numerous studies have reported the anxiety and depression of the survivors of HSCT, data which examine the severity of menopausal symptoms and the quality of life of patients for HSCT remain scarce (8–10). Specific scales have been developed and are well-accepted to measure the quality of life and menopausal symptoms (11). These include the Kupperman Menopausal Index (KMI) (12), Menopause Rating Scale (MRS) (13), Menopause-Specific Quality of Life Questionnaire (MENQOL) (14), Generalized Anxiety Disorder-7 (GAD-7) (15), Patient Health Questionnaire-9 (PHQ-9) (16), and 36-item Short-Form (SF-36) (17). Sourouni et al. identified and evaluated 751 articles, they revealed four holistic questionnaires that are frequently used to holistically assess the climacteric syndrome: KMI, MRS, and MENQOL. The scales vary in type of assessment, including symptoms, a rating system of severity, weighing of symptoms, resulting total rating score, and the validation status (18). The PHQ-9 has been validated as a diagnostic screening instrument for mood disorders (16), and GAD-7 has been validated as a diagnostic screening instrument for anxiety disorders (19). They have sensitivity and specificity for making criteria-based diagnoses and indicating depression and anxiety severity (20). In addition, the Short Form-36 (SF-36) is the most widely used health-related quality-of-life measure in research to date which included The Physical Component Summary (PCS) and Mental Component Summary (MCS) Scores (21).

We conducted a cross-sectional study to assess the severity of menopausal symptoms and compare the quality of life of patients who underwent HSCT with the norm group. In this study, we also compared the correlation among the questionnaires. In addition, we discussed the therapeutic modalities to relieve menopausal symptoms and maximize the quality of life.

## 2. Methods

### 2.1. Participants

We recruited women who had POF after HSCT for hematologic diseases between June 2017 and November 2019 in the gynecological endocrinology outpatient clinic of Peking University People's Hospital. During the survey, all women were required to use a mobile phone or tablet personal computer to scan a WeChat QR Code (Beijing Huaxin Chengda Technology Co. LTD, Beijing, China; a social media application software developed by Tencent) to enter the mobile questionnaire survey system; and to complete the Chinese version of the scales. The investigators were responsible for monitoring the results, and the physicians received training on the functions of the questionnaire system and the meaning of each item for implementing the survey. Women with HSCT were included in the study if they had 6 months of spontaneous amenorrhea with serum follicle-stimulating hormone levels greater than 40 mIU/mL taken 4 weeks apart. The patients who had other causes of POF were excluded. We analyzed the severity of menopausal symptoms, anxiety, and depression in people with POF after HSCT. In addition, differences on the SF-36 scale scores between the study group and norm groups were examined.

### 2.2. Questionnaires

Women answered questions about the type of hematologic disease, height, age, weight, and menstruation status. Every participant answered five questionnaires in the Chinese version, including the MRS, MENQOL, GAD, PHQ-9, and SF-36.

### 2.3. Menopause rating scale

The MRS consists of 11 items, divided into three domains: somatic, psychological, and urogenital domain (items 8–10) (22). For each item, the woman assigns a score of 0–4 for the severity of the symptom. The score of a particular domain is the sum of each item of the subscale. The total score of MRS is the sum of each domain. The KI and the MRS in the Chinese version, which has been validated and widely used in China, were utilized to describe the severity and intensity of menopause symptoms. Yang et al. have suggested that the modified KI and the MRS correlate well in Chinese women (23).

### 2.4. Menopause-specific quality of life questionnaire

MENQOL is a self-administered instrument consisting of 29 Likert scale items that evaluate the menopausal symptoms experienced in the last month across four domains: vasomotor, psychosocial, physical, and sexual domain. Results in study by Nie et al. have shown that the Chinese version of the MENQOL is a reasonable and valid tool for determining health-related quality-of-life for menopausal women in China (24).

#### 2.4.1. Generalized anxiety disorder-7

The GAD-7 was developed by Spitzer et al. in 2006 to help doctors recognize anxiety disorders. The GAD-7 consists of a self-report questionnaire that allows for the detection of GAD (19). Scores of 5–9 are mild, 10–14 are moderate, and ≥15 are severe. One study in Shanghai found that the Chinese version of the GAD-7 showed good reliability and validity in general hospital outpatients (25).

### 2.5. Patient health questionnaire-9

The PHQ-9 is a 9-item questionnaire to assess the presence of depression within the last 2 weeks. The PHQ-9 comprises nine items to diagnose depressive disorders (26). The score of 5–9 is mild, 10–14 is moderate, 15–19 is moderately severe, and ≥20 is severe depressive symptoms. Qian et al. have suggested that the sensitivity and specificity of the Chinese version of the PHQ-9 were 91 and 97% (27).

### 2.6. 36-item short-form (SF-36)

The SF-36 is a generic 36-item. These measures depend on patient self-reporting and are now widely utilized in adult patients (28). The survey contains 8 domains, including physical functioning (PF), general health (GH), role physical (RP), vitality (VT), bodily pain (BP), role emotional (RE), mental health (MH), and social functioning (SF). Possible scores range from 0 to 100, with higher scores representing better health status. The Chinese versions of SF-36 have been widely used in China, and their validation and normalization have been rigorously tested (29).

### 2.7. Statistical analysis

Demographic and clinical data are expressed as mean and standard deviation as well as frequencies and percentages. The SF-36 scores of patients were compared with that of individuals from the general population (norm group; *n* = 1,688) with a one-sample *t*-test. We compared variables using the Spearman rank correlation coefficient. We regrouped the symptoms of all the questionnaires into two domains, including somatic and psychological symptoms, to perform the correlation analyses of the subscales. A correlation coefficient (Rho) of 0.60 to 0.80 was considered strong, and Rho >0.80 was considered very strong (30). *P* < 0.05 was defined as statistically significant. To protect against Type I error, Bonferroni's correction will be used. Thus, each of the 10 planned comparisons will have to achieve *p* = 0.005 for statistical significance. SPSS 22.0 (IBM Corp., Armonk, NY) was used for the analysis.

## 3. Results

A total of 243 women were invited to participate in the questionnaire survey. Among those women, 16 were excluded due to incomplete questionnaires. In total, 227 (93.41%) patients completed the survey and were analyzed. Most participants were less than 35 years old (80.62%, *n* = 183). ALL (40.53%, *n* = 92) and AML (35.24%, *n* = 80) were the most frequent diseases (Table 1).

**Table 1:** Demographic and clinical characteristics of the participants after HSCT (*N* = 227).

**Age at screening (y)**	***n***	**Percentage**
<18	22	9.69
18–25	59	25.99
25–35	102	44.93
35–45	41	18.06
>45	3	1.32
Time after HSCT (y)	2.82 ± 2.11^a^	
**Types of hematologic diseases**	***n***	**Percentage**
ALL	92	40.53
AML	80	35.24
AA	24	10.57
MDS	19	8.37
Other	12	5.29

HSCT, hematopoietic stem cell transplantation; AA, aplastic anemia; ALL, acute lymphocytic leukemia; AML, acute myeloid leukemia; MDS, myelodysplastic syndrome.

a Mean values (SD).

Table 2 shows the severity of different menopausal symptoms on the MRS, MENQOL, GAD-7, and PHQ-9. The severity of all symptoms was “none and mild” in MRS, MENQOL, GAD-7, and PHQ-9. On the MRS, the most common clinical symptoms were irritability, physical and mental exhaustion, and sleep problems. The severest symptoms (Moderate + Severe + Extreme) were sleep problems (44, 19.38%) followed by sexual problems (53, 23.35%) and physical and mental exhaustion (39, 17.18%). Physical and mental exhaustion includes decreased libido, impaired memory, poor concentration, and forgetfulness. Moreover, our study demonstrated that 31.63% of the HSCT receivers had sexual partners. Thus, there is a higher incidence of sexually active individuals (73.82%)((11.89+6.17+5.29)/31.63). On the MENQOL, the most common symptoms were psychosocial and physical symptoms. The severe symptoms were sexual (35, 48.75%)((6.61+8.81)/31.63) followed by psychosocial symptoms (23, 10.13%). The psychosocial symptoms included dissatisfaction with their work life, feeling tension and anxiety, memory decline, doing things better than before, suffering low moods and depression, and lack of patience in staying alone. The percentage of moderate and severe scores were 11.89%(27) and 18.50% (42) in the GAD-7 and PHQ-9, respectively.

**Table 2:** The severity of menopausal symptoms among participants after HSCT.

**Questionnaire**	**Intensity of symptom [Number (%)]**				
	**None**	**Mild**	**Moderate**	**Severe**	**Extremely severe**
MRS		88 (38.77)	67 (29.52)	45 (19.82)	27 (11.89)
Hot flushes	104 (45.81)	92 (40.53)	28 (12.33)	2 (0.88)	1 (0.44)
Heart discomfort	132 (58.15)	75 (33.04)	18 (7.93)	1 (0.44)	1 (0.44)
Sleep problems	99 (43.61)	84 (37.00)	38 (16.74)	5 (2.20)	1 (0.44)
Depressive mood	121 (53.30)	79 (34.80)	21 (9.25)	3 (1.32)	3 (1.32)
Irritability	79 (34.80)	112 (49.34)	27 (11.89)	6 (2.64)	3 (1.32)
Anxiety	104 (45.81)	92 (40.53)	23 (10.13)	5 (2.20)	3 (1.32)
Physical and mental exhaustion	88 (38.77)	100 (44.05)	32 (14.10)	6 (2.64)	1 (0.44)
Sexual problems	127 (55.51)	48 (21.15)	27 (11.89)	14 (6.17)	12 (5.29)
Bladder problems	181 (79.74)	39 (17.18)	5 (2.20)	2 (0.88)	0 (0)
Vaginal dryness	133 (58.59)	47 (20.70)	23 (10.13)	12 (5.29)	12 (5.29)
Muscle and joint discomfort	132 (58.15)	70 (30.84)	14 (6.17)	10 (4.41)	1 (0.44)
**MENQOL**					
Vasomotor	173 (76.21)	44 (19.38)	4 (1.76)	6 (2.64)	
Psychosocial	107 (47.14)	97 (42.73)	17 (7.49)	6 (2.64)	
Physical	128 (56.39)	84 (37.00)	13 (5.73)	2 (0.88)	
Sexual	161 (70.93)	31 (13.66)	15 (6.61)	20 (8.81)	
GAD-7	111 (48.90)	89 (39.21)	18 (7.93)	9 (3.96)	0 (0)
PHQ-9	89 (39.20)	87 (38.33)	33 (14.54)	9 (3.96)	9 (3.96)

HSCT, hematopoietic stem cell transplantation; MRS, menopause rating scale; MENQOL, menopause-specific quality of life questionnaire; GAD-7, Generalized Anxiety Disorder-7; PHQ-9, Patient Health Questionnaire-9.

Norm group data for SF-36 were obtained from a population-based sample of over 1168 interviewed Chinese. For more details, see the reference article (31). Table 3 shows that in comparison with the norm group, the HSCT participants had higher VT scores and lower PF, RP and RE scores aged 18–45. In addition, the MH scores were lower in the HSCT group aged 18–25, the GH scores were lower in the HSCT group aged 25–45.

**Table 3:** The score of each dimension between participants after HSCT and the female norm of the same age group of the Sichuan population.

**Age**	**Dimension**	**HSCT group**	**Norm group**	**P**
18–25	Physical functioning	76.45 ± 20.13	90.20 ± 11.60	<0.001
	Role physical	48.64 ± 32.78	90.00 ± 21.20	<0.001
	Bodily pain	78.18 ± 20.59	84.60 ± 18.10	<0.025
	General health	59.31 ± 20.30	62.40 ± 17.30	<0.264
	Vitality	63.10 ± 19.06	54.00 ± 18.80	<0.001
	Social functioning	86.59 ± 29.84	85.80 ± 15.30	<0.845
	Role emotional	51.52 ± 31.32	85.90 ± 28.50	<0.001
	Mental health	62.47 ± 20.89	54.30 ± 20.50	<0.004
25–35	Physical functioning	75.89 ± 23.76	87.60 ± 14.4	<0.001
	Role physical	47.78 ± 38.34	86.40 ± 28.00	<0.001
	Bodily pain	77.06 ± 18.40	87.80 ± 14.70	<0.001
	General health	55.83 ± 19.74	61.70 ± 18.00	<0.006
	Vitality	62.33 ± 20.58	54.50 ± 16.80	<0.001
	Social functioning	86.67 ± 30.72	86.70 ± 15.50	<0.992
	Role emotional	45.19 ± 35.80	86.00 ± 30.00	<0.001
	Mental health	60.58 ± 20.55	58.80 ± 19.70	<0.414
35–45	Physical functioning	64.05 ± 25.52	80.80 ± 20.70	<0.001
	Role physical	46.62 ± 35.43	86.40 ± 28.00	<0.001
	Bodily pain	68.08 ± 24.09	81.70 ± 20.50	<0.001
	General health	42.51 ± 22.39	56.10 ± 20.30	<0.001
	Vitality	59.46 ± 22.29	48.90 ± 20.80	<0.007
	Social functioning	72.64 ± 27.30	83.20 ± 17.80	<0.024
	Role emotional	45.95 ± 29.78	84.20 ± 32.50	<0.001
	Mental health	55.89 ± 21.05	56.90 ± 20.80	<0.772

HSCT, hematopoietic stem cell transplantation.

Table 4 shows the correlation among the questionnaires evaluated in this study. No strong correlations (Rho > 0.80) were observed between all the questionnaires in the study. On the physical dimension, the strongest correlations were those observed between MRS and other questionnaires, particularly between MRS and MENQOL (Rho U 0.644; *P* < 0.001). In contrast, the weakest correlation was observed in comparisons involving SF-36: SF-36 vs. MRS (Rho U −0.548; *P* < 0.001); SF-36 vs. MENQOL (Rho U −0.569; *P* < 0.001). On the psychosocial dimension, the strongest correlations were those observed between PHQ-9 and other questionnaires: PHQ-9 vs. SF-36 (Rho U −0.740; *P* < 0.001); PHQ-9 vs. GAD-7 (Rho U 0.754; *P* < 0.001).

**Table 4:** Correlations among Quality of life scales of participants after HSCT measured by the Spearman rank correlation coefficient.

**Subscale**	**Questionnaire**	**MRS Rho(P)**	**MENQOL Rho(P)**	**SF Rho(P)**	**GAD-7 Rho(P)**	**PHQ-9 Rho(P)**
Physical	MRS	1	0.644 (<0.001)	-0.548 (<0.001)		
	MENQOL	0.644 (<0.001)	1	−0.569 (<0.001)		
	SF	−0.548 (<0.001)	−0.569 (<0.001)	1		
Psychosocial	MRS	1	0.723 (<0.001)	−0.631 (<0.001)	0.695 (<0.001)	0.695 (<0.001)
	MENQOL	0.723 (<0.001)	1	−0.670 (<0.001)	0.583 (<0.001)	0.649 (<0.001)
	SF	−0.620 (<0.001)	−0.670 (<0.001)	1	−0.677 (<0.001)	−0.740 (<0.001)
	GAD-7	0.631 (<0.001)	0.583 (<0.001)	−0.677 (<0.001)	1	0.754 (<0.001)
	PHQ-9	0.695 (<0.001)	0.649 (<0.001)	−0.740 (<0.001)	−0.754 (<0.001)	1

HSCT, hematopoietic stem cell transplantation; MRS, menopause rating scale; MENQOL, menopause-specific quality of life questionnaire; GAD-7, Generalized Anxiety Disorder-7; PHQ-9, Patient Health Questionnaire-9.

## 4. Discussion

This study aimed to assess the severity of menopausal symptoms and the correlation among different quality of life questionnaires and compare the quality of life of patients who underwent HSCT with the norm group in order to facilitate personalized and directed therapeutic intervention for patients. Patients reported significantly mild climacteric symptoms measured by MRS and MENQOL; however, they reported significantly worse role physical, physical functioning, bodily pain, role emotional quality of life than the norm group of the same age.

Previous reports suggested that sexual dysfunction and infertility are among the most common long-term issues after allogeneic HSCT in women (32). Studies by Klasa et al. showed that the incidence of chronic GVHD of the anogenital zone (cGVHDgyn) was 29% in female survivors after allogeneic hematopoietic cell transplantation (33). A recent study from the Seattle group found that 76% of the women who underwent HSCT reported changes in sexual life (34). In our study, there is a similar incidence of sexual problems in sex-experienced participants (73.82%). It is reported that early use of topical estrogen reduced dyspareunia, lowered the incidence rate of cGVHDgyn, and significantly improved vaginal dryness. In China, hormone therapy is not of interest due to the mild menopausal symptoms, and the HRT rate is very low (35). A study published in 2008 showed that the current rate of HT use in Beijing perimenopausal women was 1.4% (36). Therefore, further HT education among physicians and patients is needed to improve acceptance, improve the quality of life, and reducing long-term complications of patients after HSCT. Sleep disruption is a distressing, common, and underestimated problem among HSCT recipients. In a recent study, more than half of HSCT patients had poor sleep quality, and sleep problems may peak 1 month after transplantation and persist for up to a year after the patient has returned home (37). The prevalence of sleep problems in our study was 19.38% which was inconsistent with some previous studies. One reason for this difference might be that there was a long period after transplantation in this study (1–5 years). The first step to addressing sleep problems is to assess these symptoms regularly. This can be assessed by several methods, including assessment by a nurse, electronic assessment before an outpatient appointment, objective sleep data obtained from actigraphy, or patients' activity trackers (38, 39). The second step is to detect whether a patient has sleep disturbances-related organic dysfunctions such as sleep apnea which can be managed by continuous positive airway pressure (40). Moreover, patients' breathing-related sleep issues may benefit from psychological intervention. Third, patients might benefit from exercise. A recent review showed that aerobic exercise, resistance training, and mindfulness-based exercise improve sleep disruption, depression, anxiety, cardiopulmonary function, and quality of life in cancer survivors (41).

In addition to menopausal symptoms, we also assessed the quality of life of the patients. Although the field of QOL varies depending on the assessment tools, it is generally accepted that QOL includes emotional functioning, physical functioning, role functioning, social functioning, and overall QOL (42). The study by Andrykowski et al. showed that 5 years after HSCT, 18% of patients were severely restricted in physical function (43). When full recovery is expected, transplant survivors still show mild to moderate physical impairment 5–10 years after transplantation compared with cancer-free individuals. As a result, despite persistent physical limitations, many patients will return to a similar level of physical function, emotional functioning, and social functioning after HSCT as before, but at a lower level than cancer-free individuals (42, 43). Despite previous studies, our study found that the post-transplant population scored higher vitality than the same-age normal people. Increased vitality may be associated with youth, illness, non-school going, lack of job stress, easy lifestyle, and family support. In addition, MH scores are higher in HSCT in 18–25 years. Studies have shown that there is a positive correlation between the social support system and the mental health of organ transplant patients. Generally speaking, HSCT patients are a special group of people who are easy to receive care from all sides and have a good social support system. Community medical staff will follow up regularly and provide psychological counseling to help patients change their bad emotions. In addition, the hospital will regularly organize patient exchange activities to improve the mental health of patients.

Different scales contain different domains (physical, psychological, etc.). Therefore, we divided the scale into different dimensions for comparison and compared the correlation between them. It is known that patient after HSCT is a special group of people with premature ovarian failure. Our previous studies have shown that this group of people has low symptom scores on conventional scales and does not require treatment (44). We want to find the most appropriate scale that can reflect the status of patients after transplantation more comprehensively and more representatively. However, no strong correlations (Rho > 0.80) were observed between all the questionnaires in the study, indicating that each questionnaire has a special place and cannot replace each other.

This may be associated with different major concerns of the scales, which include menopausal symptoms, physical and mental health, anxiety, and depressive disorders. Due to different emphases among different scales, we suggest using the SF-36 and psychological-related scales (GAD-7, PHQ-9), in addition to common menopause scales (MRS, MENQOL) for assessing the quality of life of women after transplantation. Assessment, exercise, psychotherapy, and hormone therapy are warranted to relieve menopausal symptoms. They are the preventive measures against future complications of heart disease and osteoporosis and the main treatments for maximizing the quality of life.

We encountered some limitations in our study. Future large, multi-center, prospective studies with long-term follow-up are needed to investigate the impact of disease type, conditioning regimen, hormone therapy, participant age, and time since transplant on the quality of life of female survivors after HSCT.

## 5. Conclusions

Overall, menopausal symptoms are milder in female patients after HSCT. There is no single scale that comprehensively assesses the patient's quality of life after HSCT. We need to assess the severity of various symptoms in patients using different scales.

## Data availability statement

The raw data supporting the conclusions of this article will be made available by the authors, without undue reservation.

## Ethics statement

The study was conducted in accordance with the Declaration 228 of Helsinki, and approved by the Ethics Committee of Peking University People's Hospital (protocol 229 code 2018PHB085-01 and 2018-08-27 of approval). All patients signed an informed consent form before enrollment.

## Author contributions

HS alone was responsible for the content and writing of this paper, provided all surveys, and analyzed statistics. XY and CW designed the questionnaire. HL answered any questions raised by the participants. HZ guided our data collection and analysis. All authors contributed to the article and approved the submitted version.
